# Extracurricular sports activities modify the proprioceptive map in children aged 5–8 years

**DOI:** 10.1038/s41598-022-13565-8

**Published:** 2022-06-04

**Authors:** Can Wang, Jie Gao, Zhiqing Deng, Yichong Zhang, Chao Zheng, Xiaoli Liu, Irene Sperandio, Juan Chen

**Affiliations:** 1 Center for the Study of Applied Psychology, Guangdong Key Laboratory of Mental Health and Cognitive Science, and the School of Psychology, South China Normal University, Guangzhou, 510631 Guangdong China; 2 Department of Psychology and Cognitive Science, University of Trento, 38068 Rovereto, TN Italy; 3 Key Laboratory of Brain, Cognition and Education Sciences (South China Normal University), Ministry of Education, Guangzhou, China

**Keywords:** Human behaviour, Sensory processing

## Abstract

The Chinese government has recently issued the strictest ever guideline to improve the compulsory education system. The new policy aims at reducing the burden of excessive homework and supplementary tutoring, whilst promoting extracurricular activities, including sports and arts, for primary and junior middle school students. To examine the impact that this reform might have on sensory development—which is critical for higher-order cognitive functions—we assessed proprioceptive abilities in children from 5 to 8 years of age. Proprioception refers to sensations of position and motion of the body in space and is mediated by activity in somatosensory and prefrontal cortical areas. By asking participants to perform position matching tasks in the forward–backward directions, we were able to compare the proprioceptive maps of children with and without regular sports training. We demonstrate that extracurricular sports activities can modify the proprioceptive map and improve proprioceptive acuity and stability in school-aged children.

## Introduction

In China, parents and students have been complaining that young children spend too much time on homework, while losing precious free time for sport, recreation, and play. The most recent guideline issued by the government is aimed at reducing the pressures of excessive homework and after-school tutoring for primary and junior middle school students by limiting the teaching time (both online and offline) and homework, and by supporting sports and arts activities. The effects that these strict regulations might have on children are still not clear.

Previous research has mainly focused on how physical exercise and sports can improve mental health^1,2^ and motor skills^3,4^ in children. The Centers for Disease Control and Prevention (CDC) states that physical activity can lead to benefits for physical health: from improved weight status to increased cardiorespiratory and muscular fitness, from improved bone and joint health to reduced risk of cancer and diabetes^5–7^. Moreover, it promotes motor coordination and balance^8,9^.

But do sports also contribute to the development of brain functions? We addressed this question by examining a basic brain function, namely sensory perception. Sensory perception involves the detection and recognition of sensory signals that originate from different modalities, including visual, auditory, tactile, proprioceptive inputs, and can be considered as the cornerstone of cognitive and motor control. In this study, we tested how extracurricular sports training affects children’s proprioception. Proprioception is the sense of the position, movement, and action of the body in space. Proprioception sense also includes the sense of effort, force, heaviness and muscle tension^10,11^. Proprioception itself provides accurate space-related signals about our body parts so that even in the absence of vision, we are aware of the position of our limbs as well as their movement^12–16^. Importantly, proprioception can be integrated with signals from different sensory modalities, such as visual cues, to enable accurate motor control^17,18^.

Proprioceptive information originates from proprioceptor neurons distributed throughout the body especially in the locomotor system and the skin^11^. The proprioceptive system receives inputs from muscle spindles, joint receptors, and cutaneous receptors that signal the stretch and compression of body tissue, providing real-time information about body position^19^. Yet, the conscious report of proprioception relies exclusively on processing by the brain, particularly the posterior parietal cortex, somatosensory cortex, supplementary motor area and the premotor cortex^19–25^. Therefore, examining how sports training affects conscious proprioceptive performance would lead us to understand how sports activities affect the encoding and decoding of proprioceptive sensory information in our brain.

Here, we tested children aged 5–8 years with or without extracurricular sports training and compared their performance on proprioceptive tasks. Previous developmental studies have shown that there is a rapid increase in proprioceptive performance between 5 and 8 years of age followed by a slow improvement and stabilization in late childhood and adolescence^16,19,26^. Therefore, the present study will reveal how extracurricular sports training influences proprioceptive mapping during the critical period for proprioceptive development and how the guideline proposed by Chinese governments highlighting sports activities will influence the sensory development of children’s brains.

## Results

Participants were all right-handed. They were blindfolded with opaque eye patches for the whole duration of the experiment. To measure proprioceptive performance, we asked our participants to carry out two tasks involving movements in the forward–backward direction: delayed and online matching tasks. Everyday actions, such as grasping and reaching, are typically performed in front of our body along the forward–backward axis.

**Figure 1:**
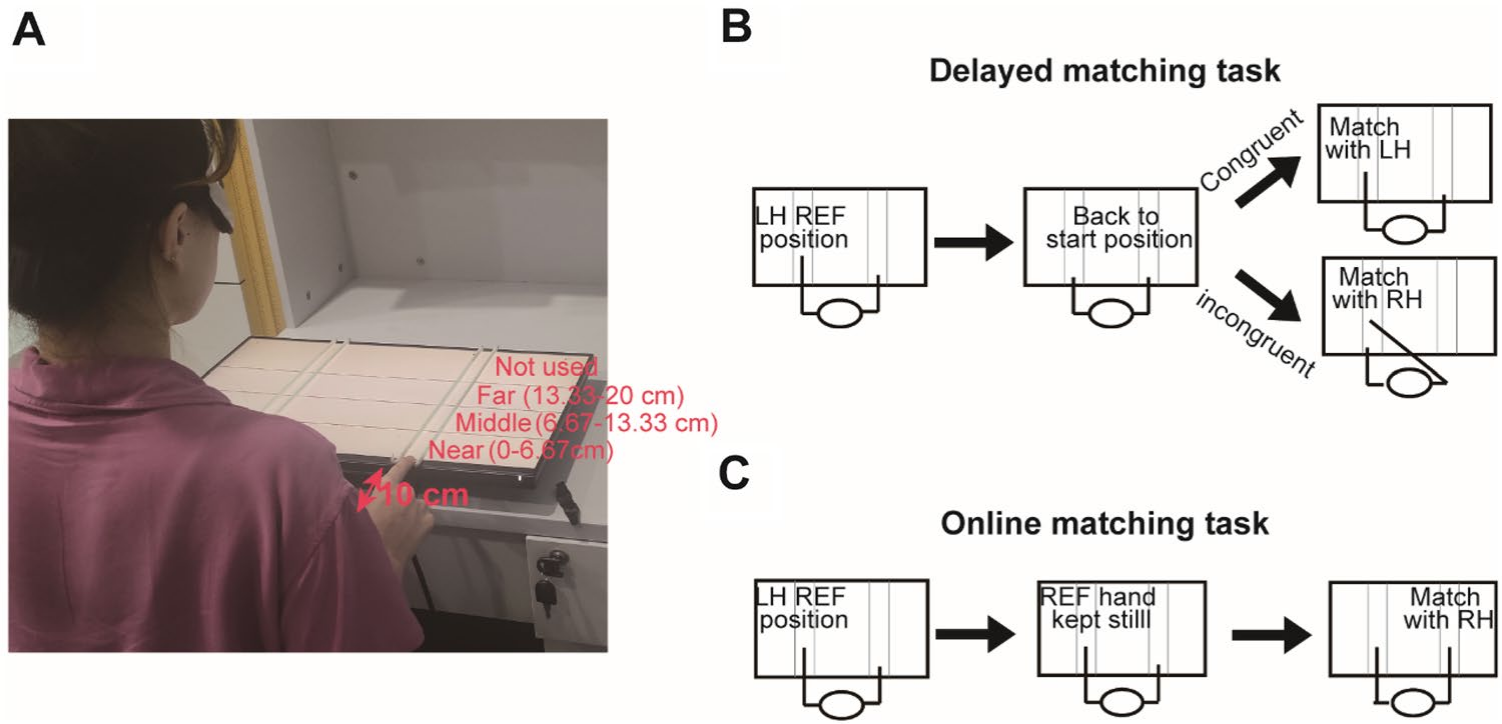
The apparatus and the protocols of the two tasks. (**A**) The distance between the participants’ bodies and the start position was 10 cm. Two pairs of white rods provided a track for participants to place their index fingers. The near, middle, and far distances were ranging from 0 to 6.67 cm, from 6.67 to13.33 cm, and from 13.33 to 20 cm away from the start position, and were all within children’s reaching distance. (**B**,**C**) The protocols of the two tasks with the left hand acting as the reference hand as an example. In both tasks, the experimenter placed the participant’s index finger of the reference hand at a specified position on the touch screen. For delayed matching task (**B**), participants first repositioned their reference hand back to the start position, and then used the same or opposite hand to reproduce (match) the reference position. For the online-matching task (**C**), participants kept their reference hand still at the reference position. Meanwhile, they used the opposite hand to match the reference position. Participants were blindfolded with opaque eye patches for the whole duration of the experiment.

The *delayed matching* task (experiment 1) tested the ability to reproduce (or match) a finger position, i.e., how well participants could point to a location where their finger was previously placed. At the beginning of each trial, the experimenter positioned the participants’ index finger of their ‘reference hand’ at one of three possible areas in the forward direction (i.e., in front of the body, Fig. 1A). Then, participants were instructed to move their reference hand back to the start position by themselves. After about 2 s, they were asked to place the index finger of their ‘matching (test) hand’ to the reference position. The reference hand could be either the left or the right hand. In the congruent condition, the same hand acted both as reference and matching hand; in the incongruent condition, both hands were involved (Fig. 1B). Unlike the congruent condition in which only one hand, i.e., only one hemisphere, was involved in the task, in the incongruent condition, participants had to retrieve the memorized position information of reference hand and then matched it with the contralateral hand. In other words, interhemispheric interactions would be involved in the incongruent condition. Therefore, by comparing the performance between congruent and incongruent conditions, we would be able to examine how interhemispheric interactions affect proprioceptive performance.

In the *online matching* task (experiment 2), again the participant’s reference hand was passively placed by the experimenter to a position (i.e., reference position) at the start of the trial. Then, participants were required to keep their reference hand still while they reproduced the finger position of their reference hand using the opposite hand. The reference hand could be either the left or the right hand (Fig. 1C). Note that proprioception from the reference position was always available during this task. As such, the online matching task measured online interhemispheric communication and calibration of position signals.

In order to simplify the analysis of the proprioceptive maps, the workspace (i.e., the area on the touchscreen where the reference finger could be placed) was divided into three areas: near, middle or far (Fig. 1A). Prior to testing, participants were invited to extend their arm out in front of them to ensure that they could reach the furthest position on the screen comfortably. All conditions were tested in all three distances. One advantage of our study is that we did not test proprioception repeatedly in several fixed positions. Instead, the reference position was randomly selected in areas specified by the experimenter, which enabled a systematic map of proprioception in the peripersonal space.

**Figure 2:**
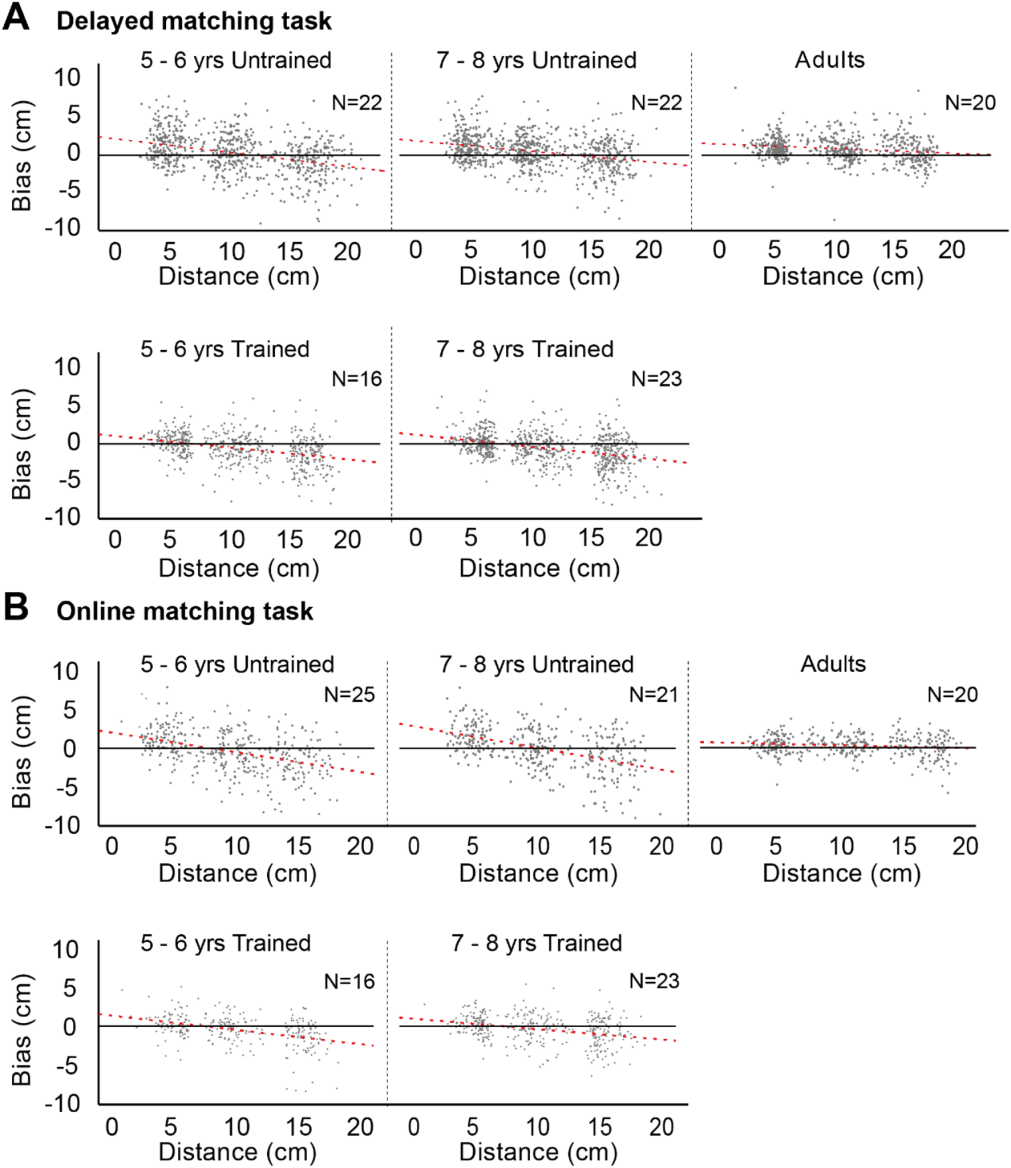
Scatter plots of the bias on all trials for delayed and online matching tasks for each group of participants. Proprioceptive bias of untrained and trained children obtained for delayed (**A**) and online (**B**) matching, respectively. Results of adults are also included for comparison. Each dot corresponds to the bias of each trial. Trials in all conditions were included. The red dashed line denotes the regression line that best fits the data in each panel. The solid black line denotes no bias.

We aimed to reveal age-related changes in the proprioceptive map and if this map could be influenced by physical activity. To this end, proprioceptive bias, quantified as the difference between reference and matching positions, was used to evaluate participants’ proprioceptive performance. A positive bias indicated that the matching position was further away (forwards) from the body than the reference position (i.e., an over-estimation), whereas a negative bias indicated that the matching position was closer to the body than the reference position (i.e., an under-estimation). Finally, the variance of the matching bias was also analyzed as a measure of proprioceptive stability. The scatter plots in Fig. 2 show the results for all age groups in both tasks. Each dot corresponds to the bias in one trial for one participant.

### Children’s proprioceptive bias in the forward direction: the “towards-middle” effect

**Figure 3:**
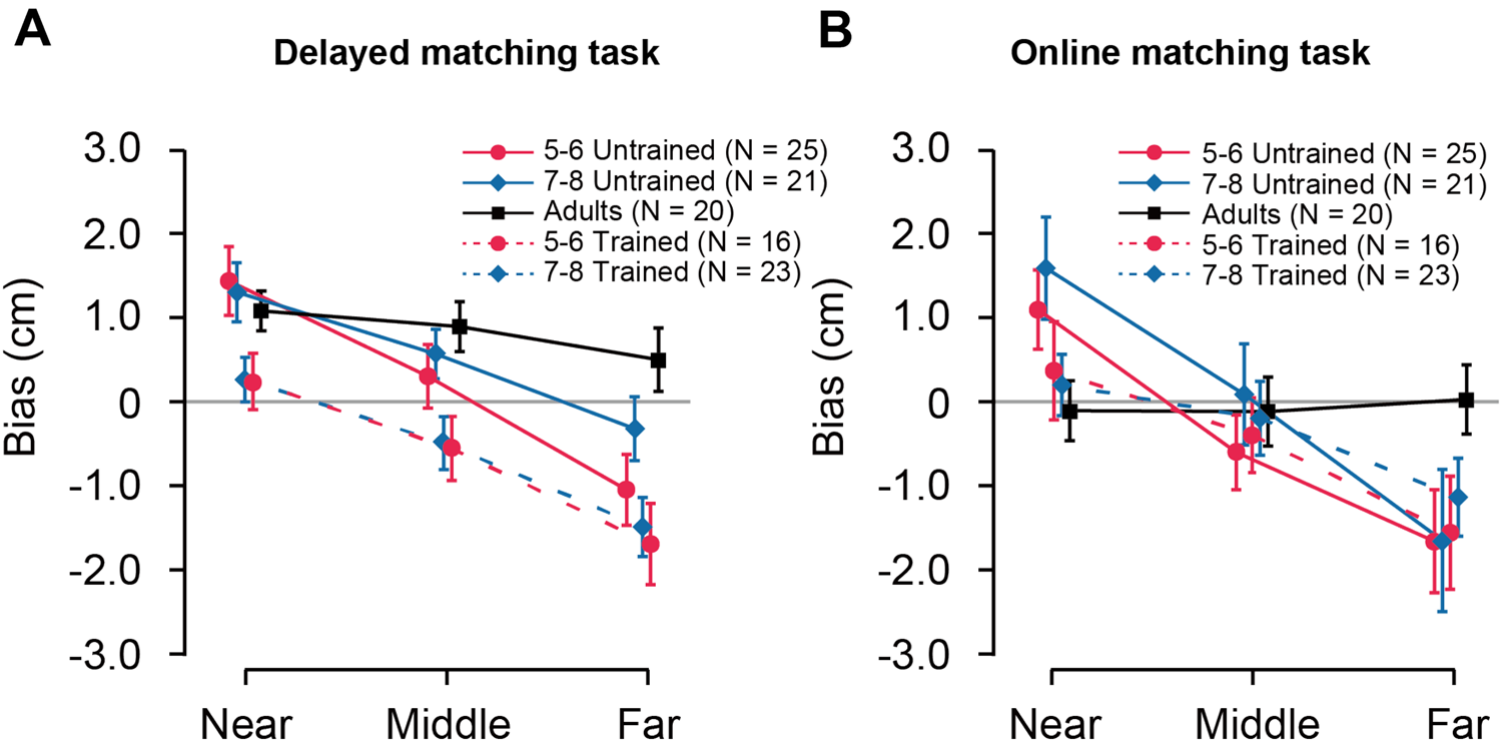
Results of bias in the delayed and online matching tasks for all groups of participants. (**A**) Bias of adults and children with (trained) or without (untrained) extracurricular sports activities and adults in the delayed matching task. (**B**) Bias of adults and children with and without extracurricular sports activities in the online matching task. Because the main effects of Reference hands and Congruency were not significant (both p > 0.6), the biases were averaged across reference hands and congruency. Error bar represents 95% confidence interval.

The delayed-matching task measured how well participants could repeatedly point to the position where their finger was previously placed. A 2 [Reference hand (left vs. right)] × 2 [Congruency condition (test hand was congruent or incongruent with reference hand)] × 3 [Distance (near vs. middle vs. far)] × 3 [Age group (5–6 years old vs. 7–8 years old vs. adults)] design was adopted. Because the main effects of Reference hands and Congruency were not significant (both p > 0.6), the biases were averaged across reference hands and congruency. Figure 3A shows the bias averaged across participants for each distance and age group.

Visual inspection of Fig. 3A suggests that the bias becomes more positive as the age increases. In other words, older participants were more likely to point to a position further away from the body than it actually was. This observation is supported by the statistical results. Specifically, 5–6-year-old had a positive bias only in the near distance (t_(21)_ = 5.94, p < 0.001), a non-significant bias (t_(21)_ = 1.33, p = 0.198) in the middle and a negative bias in the far distance (t_(21)_ = − 3.93, p = 0.001), which suggests that children of this age group tended to reproduce their finger position towards the middle (“towards-middle effect”).

The 7–8-year-old group reported a significant positive bias in the near and middle positions (near, (t_(21)_ = 6.13, p < 0.001); middle, t_(21)_ = 2.88, p = 0.009), but no significant bias in the far position (t_(21)_ = − 1.25, p = 0.224), which suggests that the “middle” of the “towards-middle effect” was shifted to the far position.

For adults, there were significant or marginally significant positive biases in all distances (near: t_(19)_ = 6.52, p < 0.001; middle: t_(19)_ = 4.67, p < 0.001; far, t_(22)_ = 1.96, p = 0.065), which suggests that adults tended to over-estimate finger positions. One possible explanation for the positive bias in adults is that the three distance conditions were defined based on children’s arm length, and therefore even the “far” distance would still correspond to “near” distance for adults. That is why the positive bias in all distances for the adult participants is equivalent to the positive bias in the near distance observed for the child participants.

Overall, these results revealed a change in proprioceptive map as a function of age, such that when reproducing a finger position, participants tended to report a previous position in the near or far locations towards the “middle”, and the “middle” distance increases with age, i.e., the length of arms.

Although the delayed matching task has been widely used in the literature to test proprioceptive accuracy, such a task necessarily involves a memory component as participants are asked to reproduce a previous finger position. The online matching task, instead, does not require memory processes as proprioceptive signals from the reference hand are always available and it allows to explore how well individuals can use interhemispheric information to guide the location of the opposite hand. We, therefore, tested whether or not children exhibited the same “towards-middle” effect when they used the index finger of the contralateral hand to match the position of the index finger of the reference hand. A 2 [Reference hand (left vs. right)] × 3 [Distance (near vs. middle vs. far) × 3 [Age group (5–6 years old vs. 7–8 years old vs. adults)] design was adopted.

Figure 3B shows the bias averaged across participants for all distance conditions and age groups. Consistent with the results of delayed matching, 5–6 years old showed a positive bias in the near distance (t_(24)_ = 3.79, p = 0.001) and a negative bias in the middle and far distances (middle: t_(24)_ = − 2.30, p = 0.03; far, t_(24)_ = − 4.96, p < 0.001). In other words, the “towards-middle effect” was replicated for cross-hand online position matching. A similar effect was observed in the 7–8-year-old group (near: t_(20)_ = 7.40, p < 0.001; middle: t_(24)_ = 0.004, p = 0.99; far, t_(22)_ = − 3.47, p = 0.002). This result is not consistent with delayed matching in which 7–8 years old had a positive bias in the middle but no bias in the far distance. Adults showed no bias in all distances (all p > 0.53) during online matching, demonstrating that proprioception is highly reliable when online information is available.

To sum up, although the proprioception map varied with task demands, a “towards-middle effect” was always reported in children without extracurricular training. The different findings obtained from the delayed and online matching tasks suggest that previous studies using delayed matching may have underestimated the acuity of proprioception^12,27^.

### Children’s proprioceptive bias in the forward direction: the influence of extracurricular sports training

To test how sports training affects the proprioceptive mapping, two new groups of 5–6 and 7–8 years old were recruited for experiment 3. All child participants had extracurricular professional training for at least 1 h per week for more than a year in sports such as basketball, football, or dancing. The sport type and training duration of each participant are listed in Tables S1 and S2 separately for each age group. To compare the proprioceptive maps of participants with and without regular physical activity, we focused our analysis on the main effect of training on proprioceptive bias.

For delayed matching, there was a significant main effect of Training on bias (F_(1,73)_ = 20.98, p < 0.001, η^2^_p_ = 0.223). Post-hoc pairwise comparisons revealed a significant difference in bias between the trained and untrained groups at the near distance (p < 0.001). While both age groups of untrained participants had a significant positive bias at the near distance (both p < 0.001), there was a non-significant bias at the near distance for both age groups of trained children (both p > 0.056). In the middle and far distances, interestingly, as shown in Fig. 3A, the bias of both age groups of trained children generally became more negative as a function of distance (dashed lines shift downwards), which demonstrates that children with sports training tended to perceive the finger position as closer to their body than the actual position, a trend that runs in the opposite direction to the effect of age (i.e., solid lines shift upwards with age). This shows that practicing sports for more than 1 year, even if unrelated to the current task, can improve proprioceptive acuity in the near distance and induce changes in the proprioceptive map across all distances.

For online matching, the main effect of Training on bias did not reach significance (F_(1,77)_ = 1.39, p = 0.24, η^2^_p_ = 0.018). However, the interaction between Training and Distance was significant ((F_(1.64,126.6)_ = 7.64, p = 0.003, η^2^_p_ = 0.09); Fig. 3B). Post-hoc pairwise comparisons showed a significant difference between trained and untrained groups only in the near distance (near, p = 0.009; middle, p = 1.00; far, p = 0.916), which demonstrates that sports training could still improve proprioception at the near distance even when online proprioceptive information was available.

Overall, sports training showed two effects on the bias. First, it improved proprioceptive accuracy in both tasks at the near distance. Second, it changed the proprioceptive map for the middle and far distances in a way that children reproduced the finger position closer to their body.

### Children’s proprioceptive bias in the forward direction: other factors

Additional analyses were carried out to examine if proprioceptive performance was also influenced by other factors, such as the congruency between the reference hand and the matching hand (using the same hand or opposite hands) or which hand acted as the reference hand (dominant or non-dominant hand).

To address this question, we analyzed the effects of Congruency and Reference hand on proprioceptive biases of the untrained participants. For the delayed matching task, the main effects of Congruency and Reference hand on bias were not significant (both p > 0.6), whereas the interaction between Distance and Congruency (F_(1.74,100.78)_ = 6.03, p = 0.005, η^2^_p_ = 0.094) was significant. Post-hoc pairwise comparisons showed no differences in bias between congruent and incongruent conditions for all three distances, although there was a trend for the difference in the near and far distances, but not in the middle distance, which probably indicates that the costs of interhemispheric interaction were larger for near or far matching distances with respect to when the matching distance was mostly comfortable (i.e., in the middle distance).

The interaction between Distance and Reference hand (F_(1.95, 112.96)_ = 4.46, p = 0.014, η^2^_p_ = 0.071) was also significant, although post-hoc pairwise comparisons revealed no significant differences in bias between left and right hand across the three distances. Overall, the bias in the delayed matching task was only slightly modulated by Congruency and Reference hand.

It should be noted that for the online-matching task, the analysis was carried out exclusively on the bias of the incongruent condition, as participants always used their contralateral hand to perform the matching task. Neither the main effect of Reference hand (F_(1, 59)_ = 0.170, p = 0.682) nor the interaction between Reference hand and other factors (Age or Distance; both p > 0.78) was significant. Therefore, the reference hand did not affect the performance during online matching.

### Stability of proprioceptive bias in the forward direction: development, training and other factors

**Figure 4:**
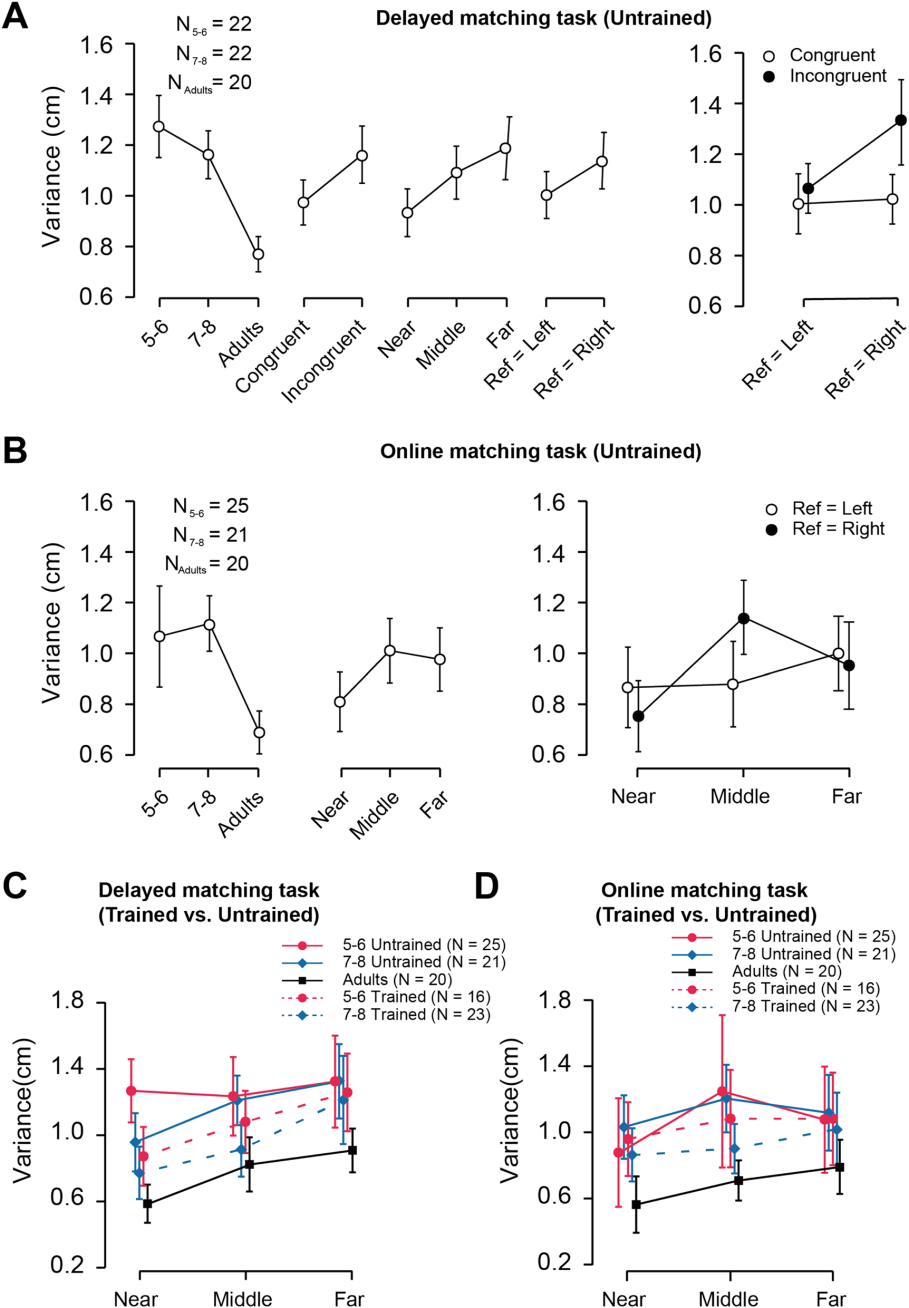
Variance in both tasks for all groups. (**A**) Left: the main effects of Age, Congruency, Distance and Reference hands on variance in the delayed matching task for the untrained groups and adults. Right: the interaction between Congruency and Reference hand on the bias in the delayed-matching task. (**B**) The main effects of Age and Distance (left panel), and the interaction between Distance and Reference hand (right panel) on the variance in the online-matching task for untrained children groups and adults. (**C**,**D**) Show the variance of adults and children with and without extracurricular sports activities in the delayed matching task and in the online matching task, respectively. Data shown were averaged over all trials, the error bar represents 95% confidence interval.

Along with bias, the stability of the matching performance was examined in untrained children as well as adults. For delayed matching, there was a significant main effect of Age on variance (F_(2, 58)_ = 15.83, p < 0.001, η^2^_p_ = 0.932). As shown in Fig. 4A, the variance of adults was smaller than that of the 5–6-years and 7–8-years groups (both p < 0.001). The main effects of Distance, Congruency, and Reference hand were also significant (Distance: F_(2, 116)_ = 8.07, p = 0.001, η^2^_p_ = 0.123; Congruency: F_(1, 58)_ = 8.19, p < 0.006, η^2^p = 0.124; Reference hand: F_(1, 58)_ = 6.67, p = 0.012, η^2^_p_ = 0.103; Fig. 4A), with smaller variances in the near distance compared to the far distance, in the congruent condition compared to the incongruent condition, and in the “reference = left” condition compared to the “reference = right” condition.

The interaction between Congruency and Reference hand was also significant (F_(1, 58)_ = 5.59, p < 0.21, η^2^_p_ = 0.088; Fig. 4A). Post-hoc pairwise comparisons revealed that the difference between congruent and incongruent conditions was significant only when the right hand acted as the reference hand (p = 0.002), which suggests that the non-dominant hand is more sensitive to the costs of the interhemispheric communication. Taken together, these results demonstrate that the stability of proprioception in delayed matching depends on age, distance, congruence, and reference hand.

For online matching task, there was a significant main effect of Age (F_(2,46)_ = 10.97, p < 0.001, η^2^_p_ = 0.323; Fig. 4B), which was manifested by a smaller variance for adults than children groups. Moreover, there was a significant main effect of Distance (F_(2,92)_ = 4.35, p = 0.017, η^2^_p_ = 0.084; Fig. 4C), whereby variance was generally smaller for near than middle and far distances. The interaction between Hand and Distance was also significant (F_(2,92)_ = 3.33, p = 0.040, η^2^_p_ = 0.067). Post-hoc pairwise comparisons showed a larger variance in the middle than the near distance only when the right hand was the reference hand (i.e., the left hand was the matching hand) (p = 0.004), suggesting that when the matching task was performed by the non-dominant hand, performance was less stable especially in the middle area.

Finally, the effect of sports training on the stability of the matching performance was investigated. For the delayed matching task, the main effect of Training (F_(1,73)_ = 8.03, p = 0.006, η^2^_p_ = 0.099) as well as the interaction between Training and Reference hand were significant (F_(1,73)_ = 5.41, p = 0.023, η^2^_p_ = 0.069). As shown in Fig. 4C right, the variance was smaller for trained than untrained groups. Post-hoc pairwise comparisons showed a significant difference between trained and untrained groups only when the right hand was the reference hand (p = 0.002), which demonstrates that sport can help the non-dominant hand to use proprioceptive information more efficiently. For the online matching task, the main effect of Training on variance was not significant (F_(1,63)_ = 1.71, p = 0.196, η^2^_p_ = 0.026; Fig. 4D).

### Asymmetry in the matching with incongruent hand

Previous studies have reported an advantage in matching accuracy when participants used their non-preferred hand, finger, limb, or foot^27–31^ (but also see^31–33^). For example, Goble and colleagues^27–29^ reported a pronounced asymmetry in delayed matching, such that the error was smaller when participants used the non-preferred limb to match the preferred limb than when they used the preferred limb to match the non-preferred limb. This was case for both right-handed and left-handed participants^28,29^.

In contrast to the advantage of the non-preferred limb (i.e., left limb for right-handed participants) reported by Goble and colleagues^27–29^, we did not find any significant difference in bias between the “Reference = Left & Match = Right” condition and the “Reference = Right & Match = left” condition for all the tested groups (all t < 2.58, p > 0.065) in the delayed matching task. With respect to variance, there was a significant difference between these two conditions only for the group of children without training, (t = 3.177, p = 0.011). Note that in opposition to Goble et al.’s findings, we observed that the variance was smaller when the right hand was used to match the position of the left hand than when the left hand was used to match the position of the right hand. In other words, the matching was less stable when the matching hand was the left hand than when the matching hand was the right hand, suggesting a right-hand advantage in the stability of matching. This was the case only for the untainted children. For adults and trained children, instead, there was no significant difference in variance between the “Reference = Left & Match = Right” condition and the “Reference = Right & Match = left condition.

A possible explanation for this discrepancy could be the different levels of task complexity between our study and Goble et al.’s previous work. In fact, in our study, participants simply matched the reference distance with their fingertip along the forward direction, whereas in Goble et al.^28^ participants were asked to match two reference positions with 20° or 40° elbow extension from the start position. Therefore, the increased processing demands along with the larger movement amplitudes involved in Goble et al.’ task with respect to ours might explain why we failed to replicate the asymmetry in accuracy.

## Discussion

This study investigated whether or not extracurricular sports training influences children’s proprioceptive map. To this end, we first systematically map the proprioceptive bias of children aged 5–8 years without extracurricular sports training using two tasks, a delayed matching task and an online matching task, and compared their performance with adults. We then used the same approach to measure the proprioceptive map of children with extracurricular sports training and compared their results with untrained children. We aimed to reveal the developmental trajectories of the proprioceptive map and if extracurricular training could contribute to children’s sensory development. Sensory processing and decoding are the cornerstone of high level cognitive and motor functions. Therefore, examining how extracurricular sports activities influences the sensory perception of children might have important implications for policy makers to improve the quality of the education system.

We found a “towards-middle” effect in children who did not practice sports on a regular basis when asked to match the location of a finger, regardless of the type of task (delayed or online matching). Specifically, near and far positions were reproduced towards the middle. To the best of our knowledge, this is the first study to report this effect. Previous studies have only showed that proprioceptive acuity improves for hand positions closer to the body^34^ or to the shoulder^35^.

Given that children showed this effect both in the delayed and online matching task, our finding cannot be simply explained by memory processes. Interestingly, adults also showed a positive bias across all distances and the bias decreased with an increase in distance, which is consistent with the ‘towards-middle effect’ if one considers that adult participants have longer arms compared to children and the far distance tested here was quite close to their body. In other words, adults may also have a “towards-middle” with the “middle” further than the furthest distance we were able to test in the current study. Further studies are needed to confirm if this effect can be observed in adults. Nonetheless, the effect disappeared during online matching. This finding highlights a developmental change possibly related to the maturation of white matter that enables a more efficient interhemispheric transfer of information during online matching, which in turn leads to a better calibration of the proprioceptive map.

Another relevant finding from the present study is that children who received extracurricular sports training for at least 1 year showed no bias at the near positions in both delayed and online matching tasks, which strongly demonstrates that sports activities can improve proprioceptive acuity of positions close to the body (up to ~ 17 cm in the forward direction). Furthermore, training modified the proprioceptive map in the middle and far distances during delayed matching. The effect of training on the proprioceptive map was different from the developmental change reported above. Whilst the bias became more positive (i.e., overestimation) with an increase in age, it became more negative (i.e., underestimation) for the trained groups than the untrained groups in the middle and far distances in the delayed matching.

In our sample there were participants with much longer training hours than others. To investigate whether or not our main results were affected by outliers, we excluded data from three participants whose training hours per week or overall training duration exceed the mean by 3 standard deviations and repeated the same analyses as described above. We found that bias and variance results did not change with respect to the results reported above. In other words, our main findings related to bias and variance were not driven by those three outliers. In addition, to investigate the effect of the amount of training on the proprioceptive performance, we calculated the Spearman’s correlation between the frequency of training (hours per week) or the total training duration (trained hours) and the bias or the variance of the trained children at each distance. Spearman’s correlation was used because the training duration followed a right-skewed distribution, rather than a normal distribution. We found that after those three participants identified as outliers were excluded, none of the correlations reached significance. This is probably due to the fact that training frequency and duration were concentrated within a small range of values (frequency < 5 h per week and duration < 200h).

It should be noted that although children aged 7–8 years had daily physical education classes in primary school, their performance did not differ much from children aged 5–6 years who were still in kindergarten. In contrast, extracurricular sports activity improved proprioceptive acuity and modified proprioceptive mapping in children from the age of five, which strongly suggests that extracurricular sports activities are more effective than physical education classes in promoting sensory improvement. This is probably due to the fact that physical education classes are characterized by a large number of students (typically > 30) where pupils may not have enough opportunities to get feedback. Moreover, the physical education taught in primary school involves mainly gross motor skills, such as running and jumping, whereas extracurricular sports activities encourage fine motor skills and provide more feedback, such as visual feedback through mirrors in the dance class.

Although the generation of proprioception comprises sensory input, central processing and motor output^36^, we speculate that the effects induced by sports training take place centrally at the level of the cerebral cortex, rather than in the joints or muscles. In fact, sensory inputs and motor outputs would have been the same for the trained and untrained children, as participants performed the matching task using a finger and their movements were restricted to the forward direction within two parallel rods.

To conclude, we report that children with at least 1 year of extracurricular sports training showed better proprioceptive performance as well as interhemispheric communication than children without extracurricular sports training. This highlights how sports activities play a critical role in the development and improvement of sensorimotor control of school-age children. The present findings have important implications for education policy makers and family education. They suggest that the most recent guideline issued by the Chinese government to reduce academic pressures and promote sports activities may facilitate the development of proprioceptive perception and hence sensorimotor control abilities of a large group of children at their critical developing period.

## Methods

### Participants

A total of 120 participants took part in this study. In experiment 1 (delayed matching task), there were three age groups of participants. Group 1 consisted of children aged 5–6 years (N = 22; 5.62 ± 0.49 years old, mean ± SD; 10 females; 12 males). Group 2 consisted of children aged 7–8 years (N = 22; 7.69 ± 0.39 years old, mean ± SD; 10 females; 12 males). None of these participants took part in any extracurricular sports activities. Group 3 consisted of young adults (N = 20; 22.40 ± 1.02 years, mean ± SD; 11 females; 9 males; 20–25 years).

There were also 3 age groups in experiment 2 (online matching task). The 5–6-years-olds consisted of 8 participants from the delayed matching task and 17 new participants (N = 25; 5.62 ± 0.49 years old, mean ± SD; 16 females; 9 males). The 7–8-years-olds consisted of 21 participants from the delayed matching task (N = 21; 7.69 ± 0.39 years old, mean ± SD; 10 females; 11 males). The adult group was the same group of participants who performed the delayed matching tasks.

For experiment 3, which tested the effects of sports training, two new groups of children (5–6 and 7–8 years old) were recruited. They all had extracurricular professional training for at least 1 h per week lasting for more than 1 year in sports such as basketball, football, or dancing. Sport type and duration of training for each participant are listed in Tables S1 and S2 separately for each age group. The 5–6 years old group had 16 participants (6.39 ± 0.54 years, mean ± SD; 9 females,7 males; training hours per week: 3.88 ± 3.685 h, mean ± SD), whereas the 7–8 years old group had 23 participants (7.95 ± 0.51 years, mean ± SD; 15 females, 8 males; training hours per week: 5.28 ± 5.02 h, mean ± SD). It should be noted that, even if all children received physical education during the school day, only those of the trained group received extracurricular professional training.

The child participants were recruited through head teachers from two different schools in Guangzhou. Head teachers were invited to distribute a recruitment flyer to parents and guardians which reported information about the study. The adult participants were recruited on a voluntary basis through posters.

All participants had normal vision and were right-handed, as determined by the hand they preferred to complete everyday activities such as handwriting. The children were recruited from public schools. None of the participants reported any neurological, psychiatric, or other major medical problems. Written informed consent was obtained from all participants. For the child participants, consent was obtained from the parent/guardian. All the participants who agreed to participate were tested and their results were reported. All participants received monetary compensation for their time. The study was approved by the Human Research Ethics Board at South China Normal University (SCNU) and the methods were in accordance with the guidelines established in the Declaration of Helsinki.

### Apparatus and stimuli

The experiment was conducted on a 24-inch Touch Screen placed on a table (resolution: 1920 × 1080; Dell). The experiment was programmed using the Psychtoolbox 3 software package (http://psychtoolbox.org/; Brainard, 1997; Pelli, 1997) in MATLAB R2020a (The Mathworks, Natick MA; https://ww2.mathworks.cn/). Proprioceptive acuity was tested along the direction away from participants’ bodies (i.e., forward direction). Four rods (5 mm in diameter) were placed on the touch screen, with each pair of rods forming a ditch to guide participants’ hand movement during matching, which ensured that participants would always match the distance along the forward direction (i.e., the direction away from their body, Fig. 1A).

Participants were seated in front of the screen. The distance between the participants’ body and the start position was 10 cm. At the beginning of the experiment, the experimenter asked participants to extend their arm out in front of them as far as they could to make sure that all participants could reach the furthest position on the screen comfortably. Three dark gray lines were presented on the touch screen, which separated the screen into four areas. Note that only the three areas closer to the participants were considered as test areas. These three areas were denoted as ‘near’, ‘middle’, and ‘far’ and corresponded to distances between 0 and 6.67 cm, 6.67 cm and 13.33 cm, 13.33 cm and 20 cm away from the start position (Fig. 1A). The 4th area was not used because our pilot study showed that some children of the 5–6 years old group had difficulties reaching that location comfortably.

### Procedure and design

Participants were blindfolded with opaque eye patches for the whole duration of the experiment. Two matching tasks were used: delayed and online (Fig. 1B,C). At the beginning of each trial, participants positioned both index fingers on the start positions. Then, a cue appeared on the screen to indicate the distance (near, middle or far). The experimenter placed the participant’s index finger of the reference hand at the cued distance areas (i.e. reference position). Two seconds later, participants were instructed to either reposition their reference hand at the start position (delayed matching) or to keep their reference hand at the reference position (online matching). Next, participants performed the matching task either with the same (i.e. congruent condition) or opposite (i.e. incongruent condition) hand with respect to the reference hand for delayed matching, but always with their opposite hand for online matching (i.e., always incongruent). The matching task required them to reproduce the reference position.

There was no time limit for the matching task. Participants did not receive any direct feedback about their performance. The coordinates of the reference and the matched positions were recorded on the touch screen and the difference in coordinates along the forward direction (i.e., along the rods) was used to define the bias.

To get familiar with the procedures, each participant performed several practice trials at the beginning of the experiment until they showed a clear understanding of the task. Participants were instructed to keep their arm relaxed and keep the index fingers of the reference and matching hands within the pairs of rods.

For delayed matching, there were 12 conditions: 2 Reference hands (left or right) × 2 Congruence conditions (test hand was congruent or incongruent to the reference hand) × 3 Reference distances (near, middle, or far). There were 3 trials per condition, for a total of 36 trials. The 36 trials were separated into 2 blocks: one with the left hand as the reference, and the other one with the right hand as the reference. Within each block, the order of trials was randomized. A short break was provided in between blocks.

For online matching task, there were only 6 conditions as the test hand was always different from the reference hand: 2 Reference hands (left and right) × 3 Distances (near, middle, or far). There were 3 trials per condition, for a total of 18 trials, which were tested in one block. Participants who took part in both experiments 1 and 2, completed the session in about 20–30 min.

The children with extracurricular sports training performed both the delayed and online matching tasks using the same design and procedures described above. The order of the two tasks was randomized.

### Statistical analysis

Spatial bias and variance were used as dependent variables to evaluate acuity and stability of proprioceptive mapping. Bias refers to the difference between the consciously perceived and the actual physical position and corresponds to a measure of mapping error. It was calculated as the difference between the mean matching position and the reference position averaged across trials. Variance was calculated as the standard deviation of bias and reflected the stability of proprioceptive matching.

ANOVAs were performed to test the main effects of factors or interactions between factors. For delayed matching task, a 4-way mixed ANOVA with Age as a between-subject factor, and the other three factors (Congruence, Reference hand, and Reference distance) as within-subject factors was performed on the bias and variance. For online matching task, a 3-way mixed ANOVA with Age as a between-subject factor, and the other two factors (Reference hand and Reference distance) as within-subject factors was performed on the bias and variance. These analyses were performed on the data from children without extracurricular sports training. To compare the performance between children with training and those without training, a between-subject factor, namely Training (with or without sports training) was included in the above-mentioned mixed ANOVAs to reveal the main effect of training and the interactions between training and other factors.

Mauchly's sphericity test was used to validate ANOVAs for within-subject factors. For variables whose distribution violated sphericity, Greenhouse–Geisser correction was performed and the results after correction were reported. Significant interactions were further analyzed using Tukey's honestly significant difference (HSD) post-hoc tests to reveal any difference between pairs of conditions. Partial eta squared (η^2^_p_) was reported to indicate the effect size for ANOVAs. Values of partial eta squared of 0.0099, 0.0588, and 0.1379 corresponded to small, medium, and large effects, respectively^37^. All the statistical analyses were performed in JASP^38^.

## Supplementary Information

Supplementary Tables.

## Data Availability

The datasets analyzed during the current study are available from the corresponding author on reasonable request.
