# Supplementary material for: Extracurricular sports activities modify the proprioceptive map in children aged 5–8 years
Source: Sci Rep. 2022 Jun 4;12:9338. doi: 10.1038/s41598-022-13565-8 (PMC9167298; doi:10.1038/s41598-022-13565-8)
Supplement: Supplementary file 1 — Supplementary Tables. [file 41598_2022_13565_MOESM1_ESM.docx]

Table S1

| **ID** | **Age** | **Exercise Type** | **Frequency/week** | **Hours Each Time** | **Total Hours /week** | **Total Duration (year)** |
| --- | --- | --- | --- | --- | --- | --- |
| 1 | 6.8 | Dance  Basketball | Everyday  Once | 1h  1h | 8h | 2.8  2 |
| 2 | 6.6 | Dance  Basketball | Everyday  Once | 2h  2h | 16h | 1.5  1 |
| 3 | 6.9 | Basketball  Roller skating | Once  Once | 1.5h  1.5h | 3h | 1  1 |
| 4 | 6.8 | Basketball | Everyday | 1h | 7h | 1 |
| 5 | 6.75 | Dance  Football | Once  Once | 2.5h  1h | 3.5h | 3.5  1.5 |
| 6 | 6.67 | Dance  Badminton | Once  Once | 1.5h  2h | 3.5h | 3  1 |
| 7 | 6.11 | Basketball | Once | 1h | 1h | 1 |
| 8 | 5.45 | Dance  Basketball | Once  Once | 1.5h  1.5h | 3h | 1  1 |
| 9 | 5 | Dance | Twice | 1.5h | 3h | 1.5 |
| 10 | 6.67 | Kung fu | Once | 1h | 1h | 1 |
| 11 | 6.33 | Kung fu  Basketball | Once  Once | 1h  1h | 2h | 1  1 |
| 12 | 5.89 | Basketball | Once | 1h | 1h | 1 |
| 13 | 6.8 | Basketball | Once | 1h | 1h | 1 |
| 14 | 6.75 | Dance  Badminton | Once  Twice | 2h  1h | 4h | 1.75  1 |
| 15 | 6.67 | Dance  Basketball | Once  Once | 1.5h  1.5h | 3h | 2.67  1.5 |
| 16 | 6 | Dance | Once | 2h | 2h | 2 |

Table S2

| **ID** | **Age** | **Exercise Type** | **Frequency /week** | **Hours Each Time** | **Total Hours /week** | **Total Duration (year)** |
| --- | --- | --- | --- | --- | --- | --- |
| 1 | 7.5 | Dance  Basketball | Once  Once | 1.5-2h  1.5h | 3-3.5h | 3  1 |
| 2 | 7 | Dance  Basketball | Twice  Once | 1h  1h | 3h | 1  1 |
| 3 | 7.5 | Dance  Basketball | Everyday  Everyday | 2.5h  0.5h | 20.5h | 1  1 |
| 4 | 7.33 | Dance | Once | 1.5h | 1.5h | 1.5 |
| 5 | 7.5 | Dance  Basketball | Once  Once | 2.5h  1.5h | 4h | 4  1 |
| 6 | 7.5 | Dance  Basketball | Three times  Twice | 0.5h  1h | 3.5h | 1  1.5 |
| 7 | 7.75 | Swimming | Twice | 1.5h | 3h | 1 |
| 8 | 7.2 | Badminton | Twice | 2h | 4h | 1 |
| 9 | 8.1 | Dance  Basketball | Three times  Twice | 3h  1h | 11h | 4  2 |
| 10 | 7.67 | Dance  Basketball | Twice  Once | 2h  1h | 5h | 3  1 |
| 11 | 8.5 | Tennis  Basketball | Twice  Twice | 1.5h  1.5h | 6h | 1  1 |
| 12 | 8.75 | Dance  Badminton | Once  Once | 1.5h  2h | 3.5h | 4  2 |
| 13 | 8.6 | Basketball | Once | 1h | 1h | 1 |
| 14 | 8.2 | Dance  Roller skating | Once  Once | 2.5h  1h | 3.5h | 2  1.5 |
| 15 | 8.5 | Badminton  Taekwondo | Once  Once | 3h  1.5-3h | 4.5-6h | 1  1 |
| 16 | 8.1 | Basketball | Twice | 1h | 2h | 1 |
| 17 | 7.8 | Dance  Roller skating  Table tennis | Three times  Once  Once | 1.5h  1h  1h | 6.5h | 3  1  2 |
| 18 | 8.5 | Dance  Basketball | Three times  Three times | 1.5h  1h | 7.5h | 4  1.5 |
| 19 | 8.45 | Taekwondo | Twice | 1.5h | 3h | 1 |
| 20 | 8.67 | Basketball | Once | 1h | 1h | 1 |
| 21 | 8.33 | Dance  Swimming  Tennis  Basketball  Badminton | Once  Three times  Three times  Three times  Three times | 1h  1-1.5h  1-1.5h  1-1.5h  1-1.5h | 13-19h | 1.5  5  3  3  3 |
| 22 | 7.45 | Dance | Once | 1.5h | 1.5h | 2.67 |
| 23 | 8 | Dance | Once | 2h | 2h | 3 |
